# *Xenopsylla cheopis* (Siphonaptera: Pulicidae) Susceptibility to Deltamethrin in Madagascar

**DOI:** 10.1371/journal.pone.0111998

**Published:** 2014-11-04

**Authors:** Sebastien Boyer, Adélaïde Miarinjara, Nohal Elissa

**Affiliations:** 1 Unité d'Entomologie Médicale, Institut Pasteur de Madagascar, Antananarivo, Madagascar; 2 Ecole doctorale Sciences de la vie et de l'environnement, Université d'Antananarivo, Antananarivo, Madagascar; Universidade Federal de Minas Gerais, Brazil

## Abstract

The incidence of bubonic plague in Madagascar is high. This study reports the susceptibility of 32 different populations of a vector, the flea *Xenopsylla cheopis* (Siphonaptera: Pulicidae), to the insecticide Deltamethrin. Despite the use of Deltamethrin against fleas, plague epidemics have re-emerged in Madagascar. The majority of the study sites were located in the Malagasy highlands where most plague cases have occurred over the last 10 years. *X. cheopis* fleas were tested for susceptibility to Deltamethrin (0.05%): only two populations were susceptible to Deltamethrin, four populations were tolerant and 26 populations were resistant. KD50 (50% Knock-Down) and KD90 (90% Knock-Down) times were determined, and differed substantially from 9.4 to 592.4 minutes for KD50 and 10.4 min to 854.3 minutes for KD90. Susceptibility was correlated with latitude, but not with longitude, history of insecticide use nor date of sampling. Combined with the number of bubonic plague cases, our results suggest that an immediate switch to an insecticide other than Deltamethrin is required for plague vector control in Madagascar.

## Introduction

Ectoparasites (including ticks and fleas) are both pests and vectors of various diseases of humans, livestock, pets, and wild animals. They can transmit diverse pathogens of medical and/or veterinary significance, including viruses, bacteria, protozoa, and helminthes [1]. Plague is one of these diseases and still remains a health problem with occasional epidemics occurring in the world. Over the last decade 82% of all cases worldwide (more than 2,000 annually, total 21,725) have been in the Democratic Republic of Congo (10,581 cases between 2000 and 2009) and Madagascar (7,182) [2], [3]. In Madagascar, 2,409 cases were confirmed between 2007 and 2011, and the country declared 67% of the worldwide cases in 2012 [4], [5].

Plague is a life-threatening infectious disease caused by the Gram-negative bacterium *Yersinia pestis* [6]–[8]. It primarily affects rodents, but can also cause outbreaks in human populations. The infection is classically transmitted in the murine population by infected fleas and the risk to human increases during epizootic periods that are associated with high rodent and flea densities. At least 80 flea species are known to carry the etiological agent of the plague, although their role in disease transmission varies [9]. For instance, the oriental rat flea *Xenopsylla cheopis* (Siphonaptera: Pulicidae), Rothschild, 1903, is considered to be the most efficient vector as well as the major vector to humans. Other flea species have been identified as vectors in East Africa, including the Islands of the South West Indian Ocean (*Ctenophtalmus bacopus*, *C. cabirus*, *Dinopsyllus lypusus*, *Pulex irritans*, *Xenopsylla brasiliensis*, *X. cheopis*) [10]. Plague was introduced into Madagascar in 1898, and then spread throughout the central highlands [4]. Two flea species are involved in the transmission of plague: *X. cheopis* and *Synopsyllus fonquerniei* (Siphonaptera, pulicidae), Wagner & Roubaud. 1932. Although *X. cheopis* is the main vector, the black rat is also frequently parasitized by the endemic flea *S. fonquerniei*, and this species contributes to the circulation of *Y. pestis* in the rural murine population [4].

Since 1947, chemical insecticides have been used to limit plague transmission during outbreaks. DDT was the first chemical insecticide used to control plague vectors in Madagascar. In 1956, the organophosphate Malathion and organochlorines were applied to plague control. At the same time, the use of the EV vaccine significantly decreased human plague cases [11]. However, the numbers of plague cases increased, particularly in the capital Antananarivo and in the coastal city of Mahajanga, after long periods of absence: 28 and 63 years, respectively [12], [13]. One of the possible causes of the increase in the incidence of human cases despite an active control campaign against plague vectors is the emergence of resistance to insecticides.

The first cases of *X. cheopis* resistance to DDT were described in 1965 in Madagascar and were first demonstrated in 1981 [14], [15]. In 1983, *X. cheopis* was reported to be resistant to Malathion, Fenitrothion and Propoxur in the field [16], [17]. Thus, the National Plague Control Program (NPCP) used Deltamethrin, a pyrethroid, to control plague outbreaks in the 1990s. After six years of use, insecticide susceptibility tests revealed *X. cheopis* resistance to Deltamethrin [18], [19]. In 2000, four populations in Madagascar were assayed for susceptibility to Deltamethrin (0.025%): one population was resistant and three populations were tolerant [20]. Pyrethroids interfere with the normal activities of nerve membranes by delaying the closing of activation gates for the sodium channel [21]. The knock-down (KD) effect describes the insect paralysis following the arrival of the molecule at the central lymph nodes. One of the major mechanisms of pyrethroid resistance involves the loss of sensitivity of the active site of the protein targeted by pyrethroids (the voltage-gated sodium channel); this is known as knockdown resistance (kdr) [22]. The existence of resistance mechanisms and their selection has been extensively studied in Diptera species (Drosophila and mosquito species) and in stored-product insects, but not in flea species. As already described for other insect vector species, *X. cheopis* in Madagascar undoubtedly displays multiple resistances against different insecticides [14], [18], [20].

Because Deltamethrin is the insecticide used by Malagasy NPCP, it is crucial to detect and evaluate the current status of flea population's susceptibility to this insecticide, in order to conduct appropriate and efficient vector control program. Here, we report an investigation of the susceptibility to Deltamethrin of *X. cheopis* in the highlands of Madagascar.

## Materials and Methods

### Ethics statements

This research in Madagascar was systematically made possible thanks to extant conventions between the IPM and local governments. The rats were caught either during an epidemic event at the request of the Ministry of Health and the World Health Organization on the basis of a National Health Priority, or during an investigative mission. In this last case, a letter was sent to the local authority and local general inspector, and to the national authority of the Ministry of Health, to explain the main objective of the field mission. The mission orders were authorized by the CSB (Centre de Santé de Base), SSD (Service de Santé de District) and Fokotany authorities. Additional authorizations were not required because *Rattus* species are considered to be pest species (especially *R. rattus* and *R. norvegicus*) and have no protected status (see IUCN and CITES lists). Traps were set only after agreement was explicitly obtained from both the village head and the field/house owner. In cultivated fields, traps were always placed at the edge of the farmed area, so that no damage was caused to crops. Rats were caught alive in wire-mesh and Sherman traps. All animals were killed by cervical dislocation. All members of the IPM involved in the fieldwork have been trained to handle and kill rodents. The study was conducted in accordance with the guidelines (http://www.pasteur.fr/ip/easysite/pasteur/en/institut-pasteur/ethics-charter) adapted to wild rodents. Animals were treated in a humane manner, and in accordance with guidelines of the American Society of Mammalogists [23]. No country-specific ethics agreement could be obtained because the country where sampling occurred and the IPM have no ethics committees relevant to animal experimentation.

Young mice used to feed flea population were from the Institut Pasteur de Madagascar (IPM) animal breeding facility. They were not purchased or donated, but were bred for this purpose.

### Trapping rats and collection of fleas

**Figure 1:**
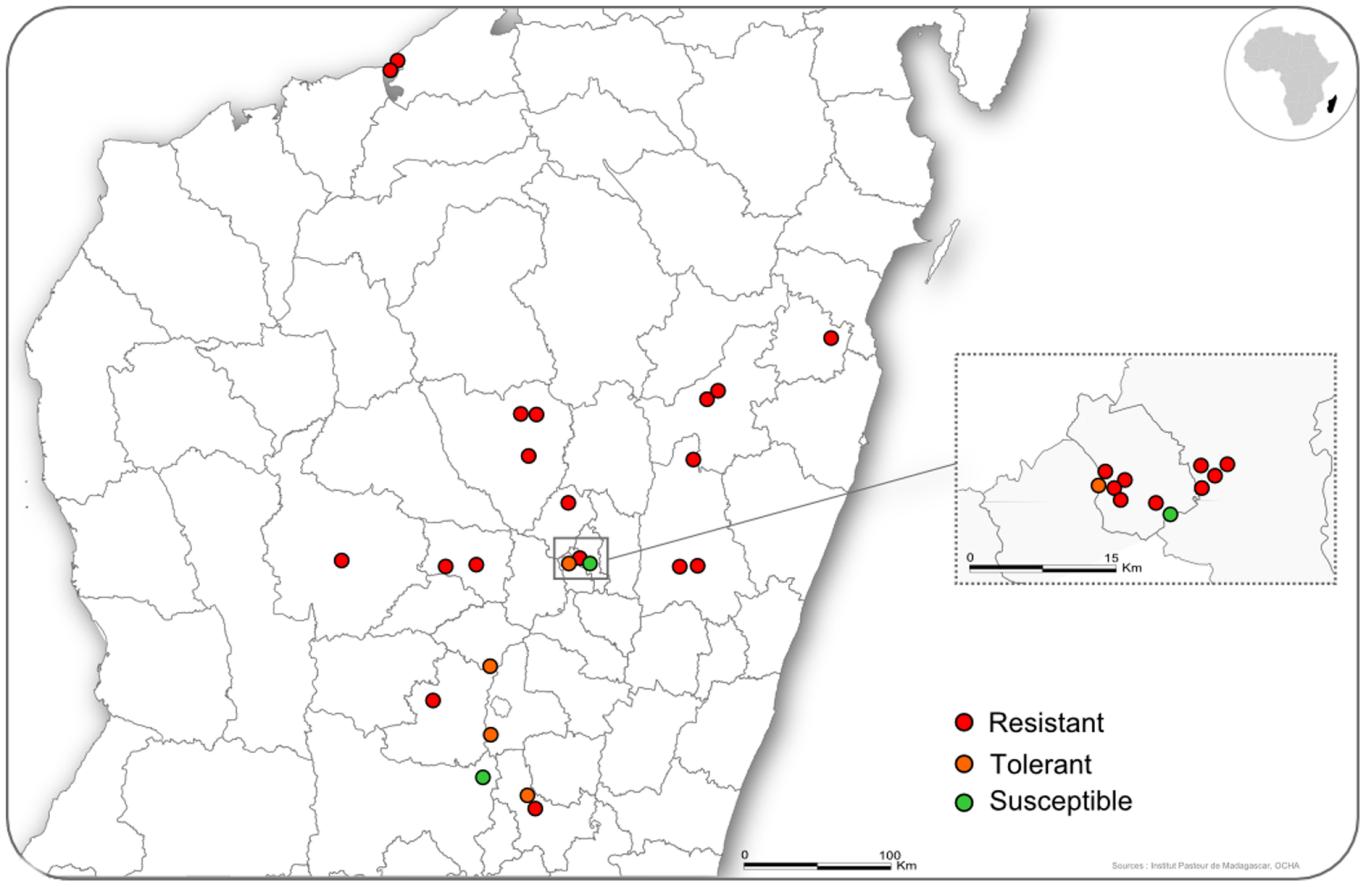
Sampling sites in Madagascar. The circles represent the villages where the flea populations were caught.

The species *Xenopsylla cheopis* was collected in 32 localities in Madagascar (Figure 1). *X. cheopis* fleas were collected on rats trapped with Sherman traps (H.B. Sherman Trap. Inc, Tallahassee, Florida) or with wire-mesh BTS traps (Besancon Technical Service, Besancon, France). For public health and sanitary reasons, all rats trapped were killed. We used trapping protocols developed and routinely used by IPM for research on *R. rattus* [24], [25].

To harvest fleas, the host was placed in a large and pale-colored basin, deep enough to prevent the fleas from escaping. Then, the fur was brushed systematically with an adapted hard-bristled brush until the fleas jumped out. The fleas were caught with a manual pump aspirator and transported alive in a box containing rice bran and food for the larvae. Fleas were identified to the species level under binocular magnifier with the Duchemin systematic key [26].

### Rearing of fleas

Fleas collected in the field were reared under laboratory conditions (22–27°C; 75–80% RH). Eggs, larvae, nymphs and adults were maintained together in clear two-liter glass jars containing 200 ml sterilized rice bran. Sixty five grams of sterilized dried ox blood, 5.5 g of dried yeast and 200 g of laboratory animal diet powder were added to the litter. The litter was changed once a year. The fleas were removed to a clean jar containing new litter, counted and divided into several jars when there were more than 200 adults; this allowed the flea population to increase more quickly. To feed the adults, each jar was provided with one live young mouse for three days every week.

### Deltamethrin bioassays

Adult fleas were bioassayed according to the WHO protocol [27]. Bioassays were carried out in 18 cm glass test tubes, covered with fine mesh cloth. Groups of ten fleas were exposed for 8 hours to paper impregnated with 0.05% Deltamethrin (1.5×6 cm; Vector Control Research Unit, Penang, Malaysia) [28]. Four replicates were carried out for each flea population. The number of dead or paralyzed fleas was counted after 10, 20, 30, 40, 60, 120, 180, 300 and 480 minutes. After the 8-hour exposure, the impregnated paper was removed (and replaced with non-impregnated filter paper). Flea mortality was scored after 24 hours. A tube with a paper impregnated with silicone oil (Vector Control Research Unit, Penang, Malaysia) was used as negative control (two replicates).

*Knock Down* 50 (KD50; the time by which 50% of fleas were knocked down) and KD90 (the time by which 90% of fleas were knocked down) were estimated from the counts during the 8 h exposure, and the susceptibility status was assessed after the 24 h observation [29], [30]: mortality rates of 98 to 100% were considered to indicate susceptibility; 80 to 98%, tolerance; and less than 80%, resistance [31]. The test was not validated, and the data not included, if the negative control mortality rate was over 10%. The mortality rate was corrected with the Abbott formula when control values were between 5% and 10%.

### Statistical analysis

The analysis was performed with R software (R version 2.15.3 2013), and Tinn-R environment (Tinn-R Editor Version 2.4.1.5 2013).

Mean KD50 and KD90 and the standard errors for each flea population were estimated with a binomial generalized linear model (glm) analysis. This glm including a probit function is a fitted model giving a prediction and a standard error at each response probability.

Analysis of Variance (ANOVA) and Tukey's b test were used to compare survival rates. Correlations between the mortality rate, KD50 and KD90 were calculated with Pearson tests. Correlations between the sampling date and the KD50, KD90 and the mortality after 24 h were also estimated with Pearson tests. ANOVA was used to test the influence of the latitude, the longitude, and the history of Indoor Residual Spraying (IRS) treatment on the three measures of sensitivity.

## Results

### Knock Down 50

KD50 values for the 32 populations were from 9.44 min to 592.35 min, and thus displayed a 63-fold difference between the flea population with the highest and that with the lowest KD50 (Table 1). Five of the 32 populations had a KD50 below 30 minutes, and six populations had KD50 values over 3 hours.

**Table 1:** Mortality values of Xenopsylla cheopis populations to Deltamethrin in Madagascar.

Sampling places	KD50^a^ (minute)		KD90^b^ (minute)		% mortality (24 hours)		GPS			
Station	Mean	SE	Mean	SE	Mean	SE	X	Y	IRS^c^ 1993–2012	Months in insectarium
Sahatany	*9,44*	*74,76*	*10,44*	*58,06*	*85,0*	*3,3*	20°02.145′	46°57.870′	9	1
Mandroseza	*11,43*	*0,89*	*18,51*	*1,48*	*100,0*	*3,9*	18°56′7,05″	47°33′1,25″	0	18
Ambanivolafotsy	*24,12*	*1,82*	*48,48*	*3,57*	47,5	1,8	20°32′24,94″	47°14′35,44″	2	2
Andavamamba	*24,66*	*4,97*	107,57	10,44	65,0	2,5	18°55′15,83″	47°30′41,35″	0	20
Ambodirano Ampefiloha	*28,59*	*2,05*	*57,14*	*4,22*	*92,5*	*3,6*	18°54′38,23″	47°29′58,69″	0	13
Soanierana	31,71	6,13	106,57	13,71	*100,0*	*5,4*	20°20′02,4″	46°54′47,7″	9	1
Ambohimiandra	33,49	3,46	91,04	7,76	65,0	2,5	18°55′44,62″	47°32′34,29″	0	17
Miadamanjaka	37,94	2,80	80,09	6,04	67,5	2,6	18°56′52,79″	46°51′57,75″	12	5
Amparihimboahangy Betafo	39,37	9,14	213,86	19,53	*85,0*	*3,3*	19°35.892′	46°38.059′	10	75
Andohatapenaka	49,52	5,29	147,03	12,36	47,5	1,8	18°54′19,63″	47°30′0,54″	0	76
Route de la gare	51,04	8,91	228,42	19,97	60,0	2,3	18°56′38,22″	48°13′49,76″	3	18
Ambohipananina	53,28	6,33	174,59	14,70	77,5	3,0	17°49′48	48°26′45,96″	4	19
Andoharano Ankazobe	56,96	3,85	115,87	8,24	37,5	1,4	18°15′0,00″	47°10′60,00″	5	3
Ankadindambo	62,80	6,61	190,83	15,41	57,5	2,2	18°54′11,08″	47°35′27,41″	10	2
Antsahatsaka	71,58	14,65	388,92	37,95	45,0	1,7	18°57′10,62″	48°16′40,84″	3	20
Ikianja	76,35	6,37	194,33	14,56	37,5	1,4	18°54′8,48″	47°34′51,38″	10	2
Tsaramasoandro	80,79	3,87	127,01	7,06	**12,5**	**0,5**	17°57′39,95″	47°13′1,20″	5	9
Tsinjorano	86,37	4,67	149,08	9,11	20,0	0,8	17°57′34,8″	47°14′52,7″	5	9
Ambatondrazaka	90,46	12,39	363,81	31,92	50,0	1,9	17°50′5,23″	48°25′29,31″	4	18
Andranofotsy	91,55	8,01	250,23	18,47	65,0	2,5	18°33′0,00″	47°28′0,00″	4	19
Tanambao	120,44	11,83	379,56	30,60	57,5	2,2	18°53′58,04″	47°35′38,65″	10	2
Iarinoro Tsarasaotra	122,02	7,60	251,45	15,76	*87,5*	*3,4*	20°26′37,76″	47°12′46,83″	6	11
Soavinarivo Betafo	125,79	8,55	284,53	18,62	35,0	1,3	19°49.452′	46°36.144′	11	77
Ambaniala	128,82	8,48	283,46	18,22	**12,5**	**0,5**	18°54′49,73″	47°34′45,24″	10	2
Andaingo	174,83	9,60	337,89	19,36	30,0	1,2	18°15′37,25″	48°15′56,15″	3	108
Andranomanalina	200,98	6,61	375,20	13,25	47,0	1,1	18°54′46,60″	47°30′34,95″	0	85
Amparaky	**230,02**	**13,62**	471,97	30,98	**2,5**	**0,1**	18°57′13,36″	46°40′20,29″	13	13
Vavatenina	**231,42**	**11,42**	415,60	22,80	17,5	0,7	17°28′3,77″	49°12′0,27″	0	78
Miandrarivo	**296,37**	**13,95**	**500,62**	**27,97**	32,5	1,3	18°56, 156′	45′59,329′	9	4
Abattoir Mahajanga	**359,34**	**27,76**	**739,93**	**69,86**	42,5	1,6	15°43′23,06″	46°19′22,75″	0	5
Tsena be Isotry	**375,39**	**22,83**	**853,58**	**63,70**	28,0	1,1	18°54′32,94″	47°31′0,65″	0	9
Tsararano Ambony	**592,35**	**51,78**	**854,29**	**93,51**	**7,5**	**0,3**	15°42′6,32″	46°19′49,48″	0	2

The most sensitive populations to Deltamethrin with the lowest KD50 and KD90 values are highlighted with italicized font. The most resistant populations to Deltamethrin with the highest KD KD50 and KD90 values are highlighted in bold. The same font code was used for the mortality percentage after 24 hours with the most sensitive population italicized, while the most resistant populations are in bold.

a KD50 represents the time, in minute, by which 50% of fleas were knocked down.

b KD90 represents the time, in minute, by which 90% of fleas were knocked down.

c IRS mean Indoor Residual Spraying, and this column represents the number of insecticide interventions the population have underwent (Max: 1 per year)

### Knock Down 90

KD90 values for the 32 populations were between 10.44 min and 854.29 min (82 fold difference). The populations with the highest and the lowest KD90 values were the same as those with the highest and lowest KD50 values (Table 1) Four of the five populations with the lowest KD50 values had a KD90 value below 1 hour. Four populations had KD90 values greater than the 8 h-exposure time with a highest value with 854.29 min.

### Mortality rate

**Figure 2:**
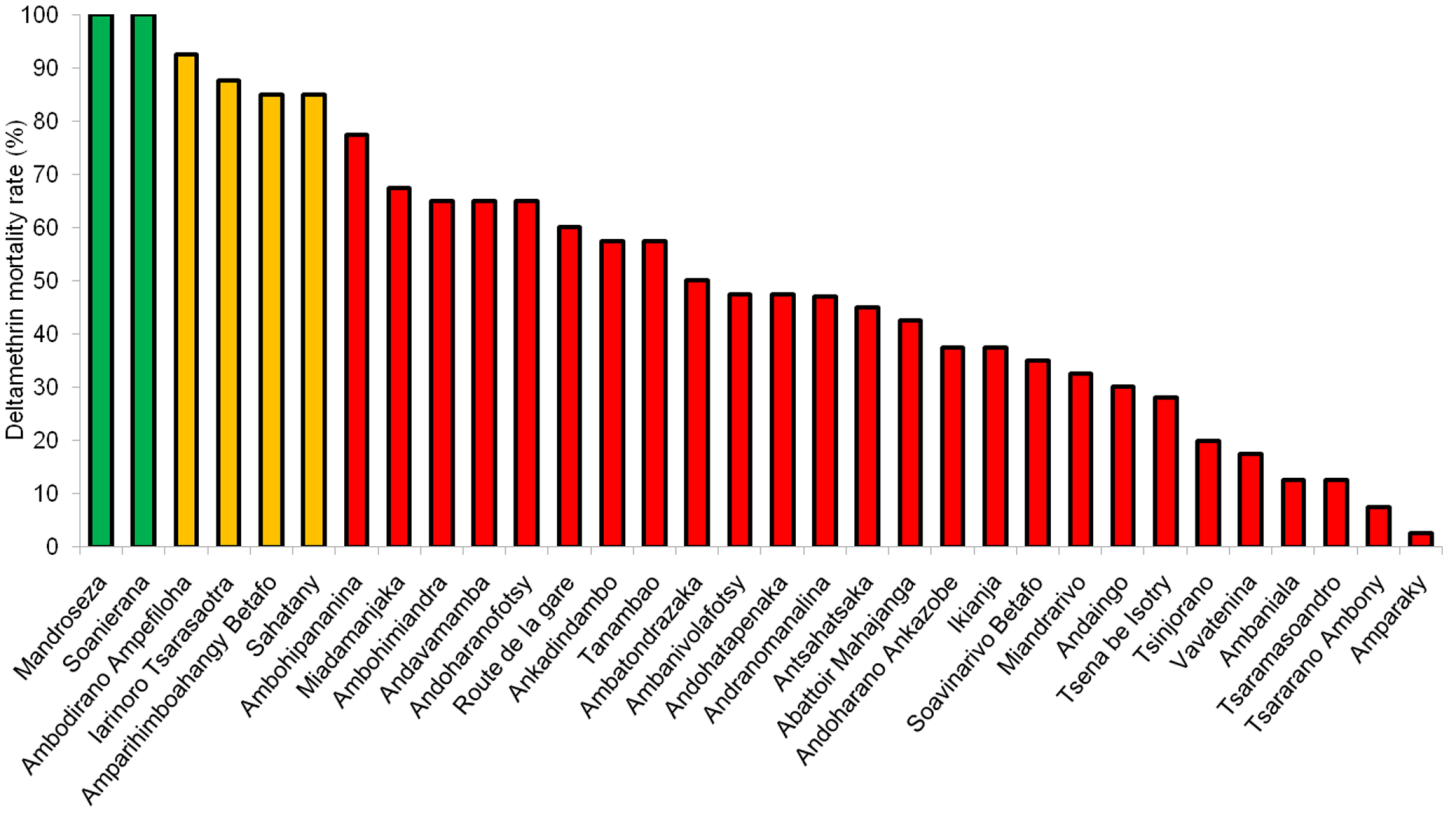
Deltamethrin mortality of flea populations sampling in Madagascar. Green bars represent sensitive populations, orange bars tolerant populations and red bars resistant populations (WHO definition).

The mortality rates of the 32 populations were between 2.5% and 100%, with an average of 50.55% (Figure 2). Two of the 32 populations were susceptible to Deltamethrin: the populations from Mandroseza and Soanierana showed 100% mortality. Four populations (Ambodirano Ampefiloha, Iarinoro Tsarasaotra, Amparhimboahangy Betafo and Sahatany) were tolerant to Deltamethrin (85–92.5% mortality), and the other 26 populations (81.25% of the populations) were resistant (2.5–77.5% mortality). The mortality rate was significantly correlated with the KD50 (r = −0.588, p<0.001) and KD 90 (r = −0.549, p = 0.0011) values.

### Correlations

No correlation was detected between the times that fleas spent in the insectarium and their KD50, KD90 or mortality (Table 2). The effect of IRS treatment on the mortality rate, the KD50 and the KD90 was not significant (Table 3). They were significant correlations between latitude and mortality, and between longitude and the KD50.

**Table 2:** Correlations results between the mortality values parameters and between the times spent by fleas in the insectariums (Pearson's correlation Test).

Tested parameters	r	95% confidence interval		t	df	p-value
24 h Survival×KD50	−0,588	−0,777	−0,301	−3,983	30	0,0004
24 h Survival×KD90	−0,549	−0,753	−0,247	−3,595	30	0,0011
KD50×KD90	0,933	0,867	0,967	14,197	30	<0.0001
KD50×Insectarium	0,010	−0,340	0,358	0,056	30	0,956
KD90×Insectarium	0,030	−0,323	0,374	0,162	30	0,8727
24 h Survival×Insectarium	−0,090	−0,425	0,267	−0,495	30	0,624

KD50 represents the time, in minute, by which 50% of fleas were knocked down, while KD90 represents the time, in minute, by which 90% of fleas were knocked down. 24 h Survival is the percentage of mortality to Deltamethrin after 24 h. And Insectarium represents the number of months during which fleas were present in the insectarium before the Deltamethrin bioassays occurred.

**Table 3:** The effect of parameters (X latitude and Y longitude: GPS localization of the sampling place; IRS: Indoor Residual Spraying which represent the number of insecticide interventions the population should underwent (Max: 1 per year) on KD50, KD90 and 24 hours survival rate of *Xenopsylla cheopis*.

	Knock-Down 50				Knock Down 90				24 h Survival rate			
	Df	F value	P		Df	F value	P		Df	F value	P	
X latitude	1	22,55	<0.0001	***	1	14,22	0,0009	***	1	9,27	0,006	**
Y longitude	1	6,91	0,015	*	1	1,78	0,194		1	0,22	0,644	
IRS	1	0,39	0,538		1	0,01	0,906		1	2,09	0,161	
latitude×longitude	1	0,03	0,870		1	0,28	0,604		1	0,46	0,505	
latitude×IRS	1	2,46	0,130		1	1,78	0,194		1	2,46	0,130	
longitude×IRS	1	2,22	0,149		1	2,60	0,120		1	2,13	0,158	
latitude×longitude×IRS	1	0,33	0,574		1	0,04	0,851		1	0,58	0,453	
Residuals	24				24				24			

Signification codes: 0 ‘***’0.001 ‘**’0.01 ‘*’0.05.

## Discussion

The KD50 and KD90 on one hand, and mortality on the other, are not indicators of the same mechanisms of resistance, and therefore not the same evolutionary selections; nevertheless, the correlations between these measures are strong enough to conclude about the Deltamethrin resistance of populations in the field. The results we report are unambiguous and raise serious concerns about flea control: 81.25% of 32 tested populations were resistant to 0.05% Deltamethrin. In 2000, only one *X. cheopis* population was found to be resistant to Deltamethrin (0.025%) [20]. Of the four populations tested in 2000, besides the one resistant population, three have become tolerant to a higher Deltamethrin concentration. Thus, the resistance has increased substantially. Analyses of pests with multiple resistances indicated that treatment with one insecticide can favor resistance to a second, different, insecticide; the example of DDT treatment influencing the resistance to other insecticides has been extensively studied and documented [32], [33]. As the resistance of *X. cheopis* to DDT has been demonstrated in the central highland of Madagascar since the early 80's, our hypothesis is that *X. cheopis* may have acquired Deltamethrin resistance after exposure to DDT treatment.

The environmental effects and/or of the anthropic pressures undoubtedly have a role in the emergence of insecticide resistance. However, latitude was the only factor found to correlate with mortality. Note that the latitude reflects a complex combination of climatic conditions (precipitations, temperature), environmental conditions such as altitude and various ecological characteristics (plant species, human social and cultural behaviors, and agricultural practices). It is therefore difficult to determine whether the relationship between latitude and insecticide resistance is due to anthropic factors or natural selection. Anthropogenic selection pressure may result from the effects of IRS, pollution, agriculture, urbanization, insecticide spraying (public health, agriculture), and the individual or collective use of pesticides. Indeed, Gratz [34] reported that mosquito vector control program mainly with DDT IRS affected the susceptibility of *X. cheopis* to this insecticide. However, in this study we find that there is no relationship between IRS treatment history (Deltamethrin, Bendiocarb), at least over the past 17 years, and flea susceptibility to Deltamethrin. Hence, at this controversy, it is difficult to assess the main factor which induces *X. cheopis* resistance to Deltamethrin, and further studies must be conducted to understand the resistance mechanisms and the associated factors. Therefore, due to the ability insects to acquire cross resistance to insecticides of the same family, other pyrethroids should not be used, and the resistance of flea populations to α-Cypermethrin, Cyfluthrin, Etofenprox, λ-Cyhalothrin, or Permethrine insecticides should be assessed before any large-scale use. It would also be valuable to understand the mechanisms of insecticide resistance in flea species, and in particular multiple resistance mechanisms.

## Conclusions

We report that only two of the 32 flea populations sampled from different locations were susceptible to Deltamethrin. Consequently, in the current context of the re-emergence of plague and the increasing numbers of human plague cases in Madagascar, Deltamethrin is ineffective against fleas. Its use in Madagascar should be stopped and the control program for plague diseases needs to change to another insecticide. Twelve insecticides will be tested in our laboratory to identify which is the most appropriate for national flea control program.
